# Acquired Vitelliform Macular Degeneration: Characteristics and Challenges of Managing Subretinal Fluid

**DOI:** 10.18502/jovr.v16i4.9748

**Published:** 2021-10-25

**Authors:** Joseph Juliano, Sagar Patel, Hossein Ameri, MD

**Affiliations:** ^1^USC Roski Eye Institute, Keck Medicine of University of Southern California, Los Angeles, CA, USA

**Keywords:** Age-related Macular Degeneration, Vitelliform Macular Degeneration, Vitelliform Maculopathy

## Abstract

**Purpose:**

To highlight diagnostic challenges in patients with acquired vitelliform macular degeneration (AVMD) with subretinal fluid (SRF) and to examine the characteristics of image findings in patients with AVMD.

**Methods:**

In this retrospective review, the electronic medical record of 22 eyes of 16 patients with AVMD was studied. The rates of SRF, drusen, pigment epithelial detachment (PED), and patient clinical information such as age, length of follow-up, and best-corrected visual acuity (BCVA) were assessed.

**Results:**

The mean age at diagnosis with AVMD was 72 years with a mean follow-up time of 29 months. Median BCVA 20/33 at presentation and 20/33 at final follow-up. Drusen was found in 13 of 22 eyes (59.1%), PEDs in 4 of 22 eyes (18.2%), and SRF in 10 of 22 eyes (45.5%) at some point during their follow-up. Of the 10 eyes with SRF, 70% were center involving, and recurrence occurred in 40%, all in the same location as the initial presentation of SRF. Three eyes received an anti-vascular endothelial growth factor injection for SRF. In 66% of cases receiving an injection, the fluid later relapsed and remitted without further injections during the course of follow-up.

**Conclusion:**

AVMD occurs in the same demographic as age-related macular degeneration (AMD) and has many common features. SRF in AVMD tends to be center involving and recurs usually in the same location as its origin. The use of anti-VEGF injections did not seem to improve SRF in contrast to the SRF seen in wet AMD. Proper differentiation of AVMD may prevent unnecessary long-term treatment with intravitreal anti-VEGF injections.

##  INTRODUCTION

Vitelliform lesions encompass a wide clinical spectrum of macular pathology. In addition to juvenile Best disease and adult onset foveomacular vitelliform dystrophy, which are associated with hereditary inheritance, there are acquired vitelliform lesions often present in the elderly. These degenerative lesions are not well characterized, and sometimes have been grouped with adult onset foveomacular vitelliform dystrophy.[1–3] In this study, we focus on acquired vitelliform lesions and use the term acquired vitelliform macular degeneration (AVMD) to focus on the spectrum of cases without a family history of the disease. Pseudovitelliform and vitelliform are terms used interchangeably to describe lesions seen in AVMD.^[4]^ While not a classic vitelliform lesion like in Best disease, in this study we will use the term vitelliform lesion to refer to the characteristic lesion appearance on fundus exam in AVMD, which is a yellow, elevated lesion usually associated with pigmentary features.^[5]^

Lesions in AVMD appear in the same demographic as dry age-related macular degeneration (AMD)^[6]^ and are often associated with drusen;^[7]^ while in AVMD the lipofuscin accumulation is within the subretinal space,^[5]^ in AMD it accumulates below the retinal pigment epithelium (RPE) and still these two entities can still be easily confused. Furthermore, subretinal fluid (SRF) can be present in AVMD^[8,9]^ which, when in the context of drusen, might be concerning for conversion of dry to wet A or, interestingly some cases of dry AMD can have SRF without evidence of choroidal neovascularization (CNV).^[10]^ Thus, in these difficult circumstances, clinicians are presented with the question: Has dry AMD converted to wet AMD, or is this SRF in the context of a vitelliform lesion? In Best disease, an abnormal electrooculogram (EOG) has been used as a reliable diagnostic tool,^[11]^ however, EOG is typically normal in AVMD.^[12]^ Practical considerations make it challenging to employ such diagnostic modalities in a busy clinic, and while imaging modalities have been employed to aid in the diagnosis such as the use of fluorescein angiogram (FA), fundus autofluorescence (FAF), or optical coherence tomography angiography (OCTA), they cannot always definitively distinguish AVMD from wet AMD. Information regarding the prevalence, natural course of AVMD associated with SRF and its management in the literature is limited. Here, we review a set of cases of patients with AVMD with particular focus on patients that develop concurrent SRF in the context of AVMD. In this study, we describe the characteristics of SRF in AVMD, the response of SRF with intravitreal injections, and suggest a general diagnostic algorithm that can be used in the clinic in order to distinguish AVMD with SRF from wet AMD.

##  METHODS

### Study Population

This study is an IRB-approved retrospective study of patients diagnosed with AVMD at a single institution over a period from 2015 to 2020. Informed consent was not required for this study. This study was conducted in accordance with the Declaration of Helsinki. The collection and evaluation of all protected patient health information was performed in a Health Insurance Portability and Accountability Act (HIPAA)-compliant manner. The study was approved by the institutional review board of the University of Southern California Health Sciences.

#### Clinical assessment

Patients were diagnosed with AVMD after meeting characteristic clinical features by combination of optical coherence tomography (OCT) and fundus exam findings. Fundus exam features included the presence of focal yellowish macular lesions and RPE changes in absence of dense pigment clumps. Characteristic OCT changes included nonhomogenous hyper reflectivity at the level of RPE that could not be classified as pigment epithelial detachment (PED) or drusen. None of the patients had a family history of the disease. All clinic visit notes were reviewed for each patient and data were obtained on age, gender, eye laterality, previous medical and ocular history, date of diagnosis, visual acuity, and type of intravitreal injection. Imaging features consisted of collecting information from clinical fundoscopic exam, fundus photos, OCT, OCTA, and FAF and FA if present. Visual acuity data were converted to LogMAR and all numerical data were reported as median or mean with standard deviation.

##  RESULTS

### Clinical Characteristics

The study included a total of 22 eyes of 16 patients with AVMD. The median LogMAR BCVA at presentation was 0.22 (20/33), and at the last final follow-up 0.22 (20/33). Five patients had diagnoses of AVMD in both eyes, eight had AVMD in the right eye, and four had AVMD in the left eye. The mean age at diagnosis was 71.6 ± 10.1 years (mean ± SD, range: 45.8–90.4 years). There were seven female and nine male patients in the study. Across all patients, the mean follow-up time was 28.5 ± 17.6 months (mean ± SD, range: 0–51.3 months). Three patients had a history of diabetes mellitus type 2 (DM2), but only one eye had evidence of diabetic retinopathy and was diagnosed as mild non-proliferative diabetic retinopathy without macular edema. Three patients had central serous chorioretinopathy (CSCR) on the differential, and 17 eyes had a prior history of dry AMD of which 1 eye had a history of wet AMD in the non-AVMD eye. Nine (9/14, 64%) patients were symptomatic with either blurry vision or metamorphopsia at diagnosis.

Three patients received an injection prior to diagnostic confirmation of AVMD. Among these individuals, the mean follow-up time was 44.5 ± 4.3 months. The BVCA of these patients at presentation was 0.13 ± 0.23 LogMAR (20/26) and at the final follow-up 0.19 ± 0.25 LogMAR (20/30). At one month prior to injection, the BCVA was 0.19 ± 0.2 LogMAR (20/30) and by one month post-injection the BCVA was 0.22 ± 0.16 LogMAR (20/33).

### Imaging Characteristics

Of the 22 eyes, 10 (45%) had a diagnosis of AVMD with concurrent SRF. One eye had SRF without a diagnosis of AVMD but had a diagnosis of wet AMD in that eye (see Patient 7 clinical course). There were four eyes with PEDs at diagnoses. No eyes with AVMD had the presence of IRF with SRF. Drusen was found in 13 eyes (59.1%) clinically, and in 15 eyes (68.2%) on OCT. Drusen was found bilaterally in nine patients on OCT. In 10 eyes with SRF, all but one eye had drusen, and three of these eyes were considered to be possibly consistent with CSCR but the diagnosis was not supported by FA. Among the patients with SRF, the total follow-up time was 35.2 ± 13.3 months. The average VA at presentation was 0.16 ± 0.13 LogMAR (20/28) and 0.19 ± 0.15 LogMAR (20/30) at final follow-up. In six eyes (6/10, 60%), the SRF was present on the first visit. SRF was bilateral in one patient. The average time to occurrence of the first SRF was 3.6 ± 5.6 (0–16) months and the average time it took for the disappearance of the first occurrence of SRF was 7.6 ± 5.6 months (0.9–14.2) months. In two eyes, the SRF did not disappear. Of these two eyes, in one eye the SRF was approximately the same size during the course of their follow-up over the period of one year, and in the other eye, it decreased in size but never fully resolved. While 9 of the 10 eyes showed decrease in the SRF over the course of their follow-up, 4 of these eyes had recurrence of the SRF, and the recurrence occurred in the same location as the initial location of SRF in all cases. Also, in three of the four eyes with PED, the presence of SRF was noted. The most common location for SRF was sub-foveal at the vitelliform lesion in 70% of eyes.

Three eyes were given an intravitreal injection for the presence of SRF. None had a history of diabetes. In one eye, the SRF resolved after one month without further recurrence over the course of follow-up over three years. In one eye, the SRF resolved within one month, but then later appeared in the same location a year later and resolved one month later without injection due to patient preference. In the third case, the SRF exhibited no response to the injection and resolved on its own over the course of one year to later relapse and remit an additional year later without further injection. Here we describe some AVMD cases in further detail to highlight the diagnostic and therapeutic challenges associated with this condition.

### Case 1: Patient No. 7

**Figure 1:**
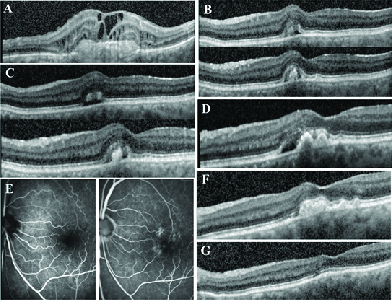
Patient number 7 clinical course. (A) OCT of the right eye demonstrating end-stage wet AMD with CNV, subretinal hyperreflective material notable for fibrosis, fluid and distorted retinal contour. (B) OCT of the left eye at presentation was notable for a vitelliform lesion with associated subretinal fluid. (C) One week after treatment with ranibizumab demonstrating persistent subretinal fluid and vitelliform lesion. (D) Four years later, new SRF appeared in the left eye adjacent to the persistent vitelliform lesion, but at that time there was no evidence of CNV by FA (E). (F) One month later, the SRF disappeared without any injections. (G) One year later, the vitelliform lesion regressed completely.

A 76-year-old female presented to clinic for blurry vision in the left eye. She had a prior history of wet AMD with disciform scar in the right eye. Her vision on presentation was 20/300 in the right eye, and 20/50 in the left eye. She reported metamorphopsia and an abnormal Amsler grid in the right eye. The anterior segment exam was notable for 2+ nuclear sclerosis in both eyes. Funduscopic examination demonstrated a macular disciform scar, and a peripheral choroidal nevus in the right eye. The left eye was notable for drusen in the macula. On OCT, the right eye demonstrated chronic changes from wet AMD notable for subretinal hyperreflective material, SRF, intraretinal fluid, and distorted foveal contour [Figure 1A]. In the left eye, there was a small pocket of SRF, with adjacent hyperreflective materials at the level of RPE, drusen, and ERM [Figure 1B]. Given the clinical picture and history of wet AMD in the right eye, the patient was treated with an intravitreal injection of ranibizumab with a presumptive diagnosis of wet AMD in the left eye.

**Table 1:** Clinical and imaging characteristics at diagnosis and last visit


**Patient number – Eye involved**	**Age at Dx/Sex**	**VA at Dx**	**Last VA**	**Concurrent retinal pathology**	**FUT (mo)**	**SRF D**	**SRF L**	**PED**	**Drusen fundus**	**Drusen OCT**
1 – OD 1 – OS	79/M	20/40 20/60	20/40 20/60	Dry AMD Dry AMD	46	– –	– –	+ –	+ +	+ +
2 – OD	45/F	20/20	20/20	–	3	–	–	–	–	–
3 – OS	57/F	20/30	20/30	Asteroid hyalosis	51	+ –	+ –	–
4 – OD 4 – OS	76/F	20/40 20/100	20/40 20/100	Dry AMD Dry AMD	0	– –	– –	– –	+ –	– –
5 – OD	66/M	20/30	20/30	–	0	–	–	–	–	–
6 – OD 6 – OS	68/F	20/25 20/30	20/25 20/30	Dry AMD Dry AMD	41	+ –	– –	– –	– +	+ +
7 – OS	76/F	20/60	20/30	Dry AMD	47	+ –	–	+ +
8 – OD	76/F	20/30	20/30	Dry AMD, ERM	32	+ –	–	–	+
9 – OD	63/F	20/20	20/20	Dry AMD	40	+ –	+ +	+
10 – OD	67/M	20/40	20/70	Dry AMD	34	+ –	–	+ +
11 – OD 11 – OS	80/F	20/40 20/50	20/50 20/40	Dry AMD Choroidal nevus OS, Dry AMD	33	+ –	+ –	– –	+ +	+ +
12 – OS	65/F	20/25	20/25	Dry AMD	47	–	–	+ +	+
13 – OD	90/M	20/40	20/30	Dry AMD	20	–	+ –	+ +
14 – OD 14 – OS	62/M	20/20 20/20	20/20 20/20	HTN retinopathy	38	– –	– –	– –	– –	– –
15 – OD	83/F	20/25	20/25	Dry AMD, Mild NPDR, No DME	24	–	–	–	–	+
16 – OD 16 – OS	73/M	20/40 20/20	20/40 20/20	Dry AMD, CSCR Dry AMD, CSCR	8	– –	– +	– –	+ +	+ +
Dx, diagnosis; M, male; F, female; VA, visual acuity; FUT, follow-up time; mo, months; SRF, subretinal fluid; D, diagnosis; L, last visit; PED, pigment epithelial detachment; OCT, optical coherence tomography; OD, oculus dexter; OS, oculus sinister; AMD, age-related macular dystrophy; Hx, history; POAG, primary open angle glaucoma; ERM, epiretinal membrane					

**Figure 2:**
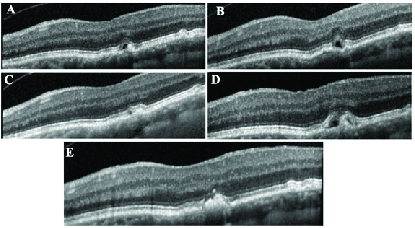
Patient number 12 clinical course. (A) OCT on presentation, three months after a previous injection of bevacizumab for presumed diagnosis of wet AMD by an outside provider. (B) Eight months after no treatment for the SRF, patient presents with new-onset SRF adjacent to a small PED. The patient was offered an injection but elected to observe; one month later, the SRF spontaneously resolved (C). (D) New-onset SRF which continued to relapse and remit over the course of her follow-up, but three years after her initial presentation (E) the vitelliform lesion condensed as well as the SRF.

**Figure 3:**
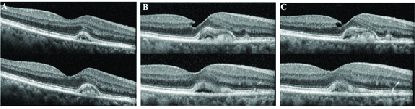
Clinical course for patient number 11. (A) Vitelliform lesion on presentation in the right eye. (B) Two years later, progression and enlargement of the vitelliform lesion with the presence of trace subretinal fluid. (C) Observed without injection over the course of three years and demonstrates stability of the vitelliform lesion and SRF.

**Figure 4:**
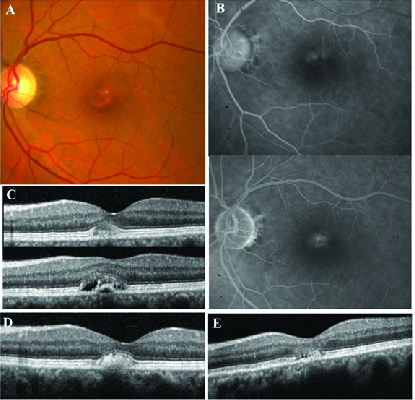
Clinical course of patient 3. (A) Vitelliform lesion in the left eye. (B) FA showing staining and leakage of the vitelliform lesion without evidence of CNV. (C) Vitelliform lesion in the left eye with associated SRF. Given the appearance of VM, the patient was observed without injection. Two years later (D), the vitelliform lesion coalesced and the SRF resolved spontaneously. (E) Five years after from presentation, the vitelliform lesion spontaneously collapsed.

One week later, BCVA was unchanged at 20/60 and OCT showed little to no change suggesting the diagnosis of AVMD consistent with OCT findings [Figure 1C]. Soon after, she received cataract extraction with intraocular lens implantation and her vision improved to 20/30. Over the course of follow-up, the vitelliform lesion stayed largely stable with relapsing and remitting SRF without further injection. Four years later, during one follow-up visit there was new SRF in the left eye which was more SRF than seen at presentation [Figure 1D]. FA was performed which showed stippled staining and leakage [Figure 1E]. Given a suspicion of wet AMD, the patient was offered a repeat trial of anti-VEGF injection, but she elected to be observed. By the next follow-up in one month, the SRF had resolved on its own [Figure 1F]. At the time of last follow-up, five years after presentation, the patient continued to have no evidence of SRF on OCT and the hyperreflective materials and foveal drusen resolved in the left eye [Figure 1G]. Her visual acuity at final follow-up was 20/500 in the right eye and 20/30 in the left eye. Throughout the course of her follow-up, despite the relapsing and remitting SRF in the left eye, she was asymptomatic and without abnormalities in her Amsler grid at each follow-up in the left eye.

### Case 2: Patient No. 12

A 67-year-old female presented for establishing care. She had a history of dry AMD in the right eye and a presumed recent diagnosis of wet AMD in the left eye for which she had received a total of three monthly bevacizumab injections. Her last injection was three months prior to presentation [Figure 2A]. At presentation, the patient had a VA of 20/30 and 20/25 with normal intraocular pressure. She was asymptomatic in the left eye with a normal Amsler grid. An evaluation of past medical records and prior OCT and OCTA images did not show features of CNV, therefore the prior diagnosis of wet AMD was put in question. The patient was followed with serial OCTs without change until eight months later when the OCT showed appearance of small pocket of SRF with an adjacent shallow PED in the left eye [Figure 2B]. The OCT changes did not support presence of wet A however, given her presumed history of wet AMD, the patient was offered a trial injection of anti-VEGF or observation. The patient opted to observe and the SRF resolved spontaneously a month later [Figure 2C]. Two years after presentation, the patient had a recurrence of SRF and appearance of hyperreflective materials [Figure 2D]. After this recurrence of SRF, the SRF was somewhat persistent but was significantly reduced in size as the hyperreflective material began to coalesce and condense at last follow-up three years after presentation [Figure 2E]. At the time of last follow-up, the vision was 20/25 in both eyes. Throughout the course of her follow-up, she denied complaints of metamorphopsia and had a normal Amsler grid.

### Case 3: Patient No. 11

A 79-year-old female presented to retina clinic for evaluation of AMD in both eyes. VA at the time of diagnosis was 20/40 in the right eye and 20/50 in the left eye. She had symptomatic complaints of “mild fuzzy vision” in both eyes over the past year with some subjective complaints of wavy lines in both eyes. The Amsler grid was abnormal focally at one spot temporally on each eye. IOP and the anterior exam was unremarkable. Her fundus exam showed dry AMD in both eyes, a choroidal nevus in the left eye and a vitelliform lesion in the right eye just nasal to the fovea [Figure 3A]. Over the course of two-year follow-up, the patient developed significant progression of the vitelliform lesion in the right eye and trace SRF within the lesion [Figure 3B]. Based on the characteristic appearance of AVMD in this patient, she was observed without injection. At the day of last follow-up, three years after her first presentation, she had a stable small SRF and a vitelliform lesion [Figure 3C] that was observed without injection. Her vision on final follow-up was 20/50 in the right eye and 20/40 in the left eye. Over the course of her follow-up, she reported occasional metamorphopsia in the right eye with a consistently abnormal Amsler grid; while in the left eye it appeared to be normal for the course of her follow-up after her first abnormal Amsler grid on initial presentation.

### Case 4: Patient No. 3

A 57-year-old male presented for retinal evaluation and establishing care. One and a half years prior to presentation, he had received an unknown injection in the left eye by a different provider. Presenting visual acuity was 20/20 in the right eye and 20/30 in the left eye. IOP and anterior exam was unremarkable. The patient complained of symptomatic distortion in the left eye and had an abnormal Amsler grid. On funduscopic examination, the patient had RPE mottling in the right eye and a vitelliform lesion in the left eye [Figure 4A]. While the patient appeared to have SRF in the left eye that could be consistent with CSR or vitelliform, by FA it appeared the pattern was less characteristic for CSR or CNV [Figure 4B]. On OCT, the right eye was unremarkable but the left eye demonstrated AVMD with the presence of SRF [Figure 4C]. Given the characteristic appearance for vitelliform lesion, the lesion was observed without injection. Over a period of two years without injection, the vitelliform lesion coalesced, and the SRF resolved spontaneously [Figure 4D]. By follow-up at five years from the initial presentation, the vitelliform lesion had collapsed [Figure 4E]. Throughout the course of the follow-up, the patient reported improvement of vision in the left eye with a final visual acuity of 20/30 (and 20/30 in the right eye) but continued complaints of metamorphopsia and abnormal Amsler grid in the left eye.

##  DISCUSSION

There is a considerable diagnostic challenge with AVMD. Given the older average age, presence of drusen in a large percentage of patients with AVMD, distinction from dry AMD is challenging. While modern multimodal imaging remains helpful, there is not a single diagnostic test or imaging modality that can provide a definitive diagnosis. Worse yet, at times subretinal fluid (SRF) accompanies AVMD lesions which presents a diagnostic dilemma to the clinician: Does this represent progression from dry to wet AMD, or simply SRF associated with AVMD? In this study we focused on acquired vitelliform lesions that presented diagnostic challenges as a result of the association of SRF with these vitelliform lesions.

The characteristic lesion appearance of AVMD on ophthalmic exam is a yellow, elevated lesion approximately 1/3 to 1 disc diameter in size^[13]^ with the presence of nonhomogenous hyperreflectivity that accumulates within the subretinal space, in contrast to the appearance of PED or drusen which accumulate below the level of the RPE. These lesions appear similar to the vitelliform lesions found in Best disease but unlike hereditary vitelliform diseases, acquired diseases appear in a much older demographic, typically around 70 years of life.^[7]^ The yellow appearance is a result of a particular amount of accumulated loose lipofuscin and photoreceptor debris.^[5]^ SRF in this entity is considered to be a result of mechanical displacement between the outer retinal layers and the RPE^[10]^ inhibiting the RPE from pumping out the liquified lipofuscin debris. Alternatively, it may represent incompetent RPE unable to move fluid into the choriocapillaris. Visual acuity tends to typically remain good at presentation despite the vitelliform lesion at the fovea. In one study of 17 eyes, among eight patients with AVMD, the median VA acuity was 20/40 at presentation, and by final follow-up 71% retained vision within one to two lines of their initial visual acuity.^[7]^ These characteristic features were similar to our study. The mean age of our patient population on diagnosis was 72 years and the median visual acuity in our study at both diagnosis and final follow-up was approximately 20/29 over an average follow-up interval of approximately 2.4 years. Although we suspect that the average visual acuity was similar to presentation because during the course of their follow-up patients underwent cataract extraction.

The appearance of these lesions, particularly in the context of dry AMD, can be concerning for progression to CNV, and ancillary imaging such as OCT and FA can be particularly useful. Other studies have noticed a presence of drusen anywhere between 33 and 60% of patients concurrently diagnosed with AVMD.^[2,14,15]^ Our patients similarly had concurrent macular degeneration which made the diagnosis challenging. Of the 22 eyes, 17 (77%) had concurrent diagnosis of dry AMD in addition to a diagnosis of AVMD. Additionally, 68.2% had drusen notable on OCT. The vitelliform lesion itself may have pigment which can be helpful in differentiating from CNV. In vitelliform lesions, the pigment is located centrally and surrounded by a hypopigmented halo. The vitelliform lesions tend to be less vascular and demonstrate decreased density of blood vessels at the superficial, deep capillary plexus and choriocapillaris^[16]^ thought to be a result of accumulation of the vitelliform material that physically displaces the capillary network. In our study, we used OCTA in some cases with SRF but failed to detect any CNV. Additionally, no eyes with AVMD were found to have concurrent SRF and IRF which further decreased the likelihood of fluid caused by CNV.

We relied predominately on funduscopic exam and OCT to assess and diagnose AVMD. Characteristically, the lesion is located in the subretinal space between the RPE and neurosensory retina^[17]^ which is where our lesions were predominately located as seen in the patient images throughout this study. We found the existence of PED in four eyes in our study (18%), and SRF in 10 of 22 eyes (46%) which was a bit higher than other studies identifying PEDs in approximately 7–8% of eyes;^[8,9]^ and another study demonstrating 21.1% of eyes had evidence of SRF associated with the acquired vitelliform lesions.^[8]^ In this study, the majority of eyes with SRF also had drusen. Notably, among all eyes but one (a patient with wet AMD), there was no SRF in the fellow eye unless a vitelliform lesion was present which suggests that the SRF was likely due to vitelliform material accretion and was unlikely to have developed due to other conditions such as diabetes or undiagnosed inflammatory/infectious diseases. We showed that the SRF tended to be located sub-foveally adjacent to the area of the vitelliform lesion, and when the fluid recurred as found in 40% of patients with SRF, it was located in the same location as on presentation.

The impact of anti-VEGF in these lesions remain unclear and current literature offers no consensus on their benefit in this disease entity. In one case report, only one injection of bevacizumab was done and over time, the SRF decreased,^[18]^ while in another case report, there was little improvement with these lesions.^[19]^ As a result of the diagnostic confusion regarding AVMD, patients often receive many anti-VEGF agents prior to finally being diagnosed with AVMD. In one study, six patients were initially diagnosed to have occult CNV from AMD and were not diagnosed with AVMD until after finishing a series of three injections of ranibizumab with little response on OCT.^[20]^ In our series, we report the results of three patients who were given anti-VEGF injections (two with bevacizumab, one with ranibizumab) because AVMD could not be differentiated from wet AMD. In this study, we predominately used the response of anti-VEGF to gauge whether the SRF accumulation was a result of progression to wet AMD or the coexistence of SRF with AVMD. There was no clear response to anti-VEGF injections, and most often the SRF resolved spontaneously without the use of injections. Among those that did have an injection, in one eye the SRF resolved, but in the other two it either exhibited no response, or relapsed and remitted even without further use of injections as illustrated by Cases 1 and 2.

We suggest that while the use of anti-VEGF may not be useful to treat these patients, a treatment trial can be useful in difficult situations where there is a suspicion of AVMD in the setting of new onset SRF. Current diagnostic modalities such as genetic testing are often impractical for the patient presenting in clinic with SRF. FA and OCTA can be useful to characterize the presence of CNV, however, they may still have difficulty detecting occult CNV. In our study, there was leakage on FA even in the absence of CNV. In these uncertain diagnostic situations, a clear discussion with the patient can help delineate appropriate management options. There are two options that can be presented: one is to monitor closely and start treatment when the SRF continues to increase and the patient becomes symptomatic. The other option is to give the patient an anti-VEGF injection and bring the patient back in one to two weeks to determine the response. If the SRF has either been unchanged or only minimally changed, the patient can be presumptively diagnosed with AVMD and monitored closely. If the fluid does resolve after the first injection, it is possible that this patient has CNV. As illustrated by case 2, however, SRF may spontaneously resolve on its own without injection, so if an injection is given and the fluid resolves, it may be a result of the natural history of relapsing and remitting SRF in AVMD rather than the effect of the injection itself. Thus, periods of anti-VEGF holidays may be helpful to distinguish between AVMD and AMD. Recurrence or worsening of fluid would be suggestive of AMD, whereas resolution or stability of fluid would be suggestive of AVMD.

In summary, the diagnosis of AVMD can be challenging given its heterogenous appearance. When SRF develops, these cases provide a diagnostic dilemma to the clinician who may suspect possible progression to wet AMD. We suggest close observation or a trial of anti-VEGF for the SRF and re-evaluation in one to two weeks. If there is no response to an injection, then consider observing the SRF rather than providing repeated monthly treatments. Further work is needed to properly differentiate the heterogenous appearance of these disease processes and the response to anti-VEGF agents in order to avoid unnecessary treatment.

##  Financial Support and Sponsorship

The authors received no financial support for the research, authorship, and/or publication of this article.

##  Conflicts of Interest

The authors declare no potential conflicts of interest with respect to the research, authorship, and/or publication of this article.

## References

[B1] Chowers I, Tiosano L, Audo I, Grunin M, Boon CJF (2015). Adult-onset foveomacular vitelliform dystrophy: a fresh perspective. Prog Retin Eye Res.

[B2] Greaves AH, Sarks JP, Sarks SH (1990). Adult vitelliform macular degeneration: a clinical spectrum. Aust N Z J Ophthalmol.

[B3] Jun I, Lee JS, Lee JH, Lee CS, Choi S, Gee HY, et al (2017). Adult-onset vitelliform macular dystrophy caused by BEST1 p. Ile38Ser mutation is a mild form of best vitelliform macular dystrophy Sci Rep.

[B4] Sabates R, Pruett R, Hirose T (1982). Pseudovitelliform macular degeneration. Retina.

[B5] Arnold JJ, Sarks JP, Killingsworth MC, Kettle EK, Sarks SH (2003). Adult vitelliform macular degeneration: a clinicopathological study. Eye.

[B6] Freund KB, Laud K, Lima LH, Spaide RF, Zweifel S, Yannuzzi LA (2011). Acquired vitelliform lesions. Retina.

[B7] Lima LH, Laud K, Freund KB, Yannuzzi LA, Spaide RF (2012). Acquired vitelliform lesion associated with large drusen. Retina.

[B8] Freund KB, Laud K, Lima LH, Spaide RF, Zweifel S, Yannuzzi LA (2011). Acquired vitelliform lesions: correlation of clinical findings and multiple imaging analyses. Retina.

[B9] Saito M, Iida T, Freund KB, Kano M, Yannuzzi LA (2014). Clinical findings of acquired vitelliform lesions associated with retinal pigment epithelial detachments. Am J Ophthalmol.

[B10] Sikorski BL, Bukowska D, Kaluzny JJ, Szkulmowski M, Kowalczyk A, Wojtkowski M (2011). Drusen with accompanying fluid underneath the sensory retina. Ophthalmology.

[B11] Deutman AF (1969). Electro-oculography in families with vitelliform dystrophy of the fovea: detection of the carrier state. Arch Ophthalmol.

[B12] KrÃ¤mer F, White K, Pauleikhoff D, Gehrig A, Passmore L, Rivera A, et al (2000). Mutations in the VMD2 gene are associated with juvenile-onset vitelliform macular dystrophy (Best disease) and adult vitelliform macular dystrophy but not age-related macular degeneration. Eur J Hum Genet.

[B13] Hanif AM, Yan J, Jain N (2019). Pattern dystrophy: an imprecise diagnosis in the age of precision medicine. Int Ophthalmol Clin.

[B14] Burgess DB, Olk RJ, Uniat LM (1987). Macular disease resembling adult foveomacular vitelliform dystrophy in older adults. Ophthalmology.

[B15] Gass J (1974). A clinicopathologic study of a peculiar foveomacular dystrophy. Trans Am Ophthalmol Soc.

[B16] Querques G, Zambrowski O, Corvi F, Miere A, Semoun O, Srour M, et al (2016). Optical coherence tomography angiography in adult-onset foveomacular vitelliform dystrophy. Br J Ophthalmol.

[B17] Benhamou N, Messas-Kaplan A, Cohen Y, Gaudric A, Souied EH, Soubrane G, et al (2004). Adult-onset foveomacular vitelliform dystrophy with OCT 3. Am J Ophthalmol.

[B18] Montero JA, Ruiz-Moreno JM, De La Vega C (2007). Intravitreal bevacizumab for adult-onset vitelliform dystrophy: a case report. Eur J Ophthalmol.

[B19] Kandula S, Zweifel S, Freund KB

[B20] Gallego-Pinazo R, Dolz-Marco R, Pardo-LÃ³pez D, Arevalo JF, DÃ­az-Llopis M (2011). Primary intravitreal ranibizumab for adult-onset foveomacular vitelliform dystrophy. Graefe’s Arch Clin Exp Ophthalmol.

